# Dr. Stanley Hunter Darlington

**Published:** 1912-10

**Authors:** 


Dr. Stanley Hunter Darlington, Buffalo, 1902, a resident of Buffalo but not listed in the medical directory, died Aug. 18, aged 35.

